# A finite element analysis study on different angle correction designs for inclined implants in All-On-Four protocol

**DOI:** 10.1186/s12903-024-04091-2

**Published:** 2024-03-13

**Authors:** Christine Raouf Micheal Ibrahim, Ahmed Sameh, Osama Askar

**Affiliations:** 1 Department of Prosthodontics, Faculty of Dentistry, Mansoura University, Eldakahlia, Egypt; 2 Production Engineering and Mechanical Design Department, Faculty of Engineering, Mansoura University, Eldakahlia, Egypt

**Keywords:** Angled multiunit abutment, Finite element analysis, Angle corrected implant

## Abstract

**Background:**

The aim of this study is to investigate, through finite element analysis (FEA), the biomechanical behavior of the built-in angle corrected dental implant versus implant with angled multiunit abutment used in All-On-Four treatment protocol.

**Methods:**

Two (3D) finite element models of a simplified edentulous mandible were constructed with two different posterior implant designs based on the All-On-Four protocol. Four implants were placed in each model, the two anterior implants were positioned vertically at the lateral incisor/canine sites. Depending on the implant fixture design in posterior area, there are two models created; Model I; the mandible was rehabilitated with four co-axis (4 mm in diameter × 15 mm in length) implants with distally built-in angle corrected implants (24-degree angle correction) .While Model II, the mandible was rehabilitated with four conventional (4 mm in diameter × 14 mm in length) implants with a distally inclined posterior implants (25 degree) and angled multiunit abutments. CAD software (Solidworks© 2017; Dassault Systems Solidworks Corp) was used to model the desired geometry. Axial and inclined Loads were applied on the two models. A Finite element analysis study was done using an efficient software ANSYS© with specified materials. The resultant equivalent Von-Misses stresses (VMS), maximum principal stresses and deformation analysis were calculated for each part (implants and prosthetic components).

**Results:**

When applying axial and non-axial forces, model II (angled multiunit model) showed higher deformation on the level of Ti mesh about 13.286 μm and higher VMS 246.68 MPa than model I (angle corrected implant). Model I exhibited higher maximum stresses 107.83 MPa than Model II 94.988 MPa but the difference was not statistically significant.

**Conclusion:**

Within the limitation of the FEA study, although angle correcting implant design is showing higher values in maximum principle stresses compared with angled multiunit abutments, model deformation and resultant VMS increased with angled multiunit abutments. The angle correcting designs at implant level have more promising results in terms of deformation and VMS distribution than angle correction at abutment level.

**Supplementary Information:**

The online version contains supplementary material available at 10.1186/s12903-024-04091-2.

## Background

Restorations supported by dental implants are chosen over removable complete dentures because they provide a more comfortable treatment option for completely edentulous patients. Furthermore, they can address issues like ill retentive dentures, instability and dissatisfaction of patients with complete dentures [1]. Implant restorations have a good survival rate and enhance patients’ quality of life specially full-arch implant-supported fixed dental prosthesis (FAFDP) which is considered to be the most preferred treatment solution for complete edentulous patients [2]. However, the success of FAFDPs is mostly depending on the treatment strategy, implant form, number, and configuration but the clinicians’ experience and skills are also important factors [3]. 

Depending on the patient’s anatomy and selected clinician’s technique, there are different implant numbers and distribution used to support an FAFDP. Since the 1990s, four or six implants have been used as a standard approach for supporting mandibular FDPs [4]. Eliasson et al. [5] proposed that the use of four implants between the mental foramina in edentulous patients is used to receive fixed restorations and can avoid encroaching inferior mental nerves. Unfortunately, due to the restricted bone volume between the mental foramina, prostheses with long span cantilevered prosthesis are required which can increase the load on the distal abutments and distal implants by up to two or three times, resulting in undesirable stress concentration and mechanical failures of the components [6]. 

Nowadays, axially inserted, and inclined implants are used as in the All-On-Four concept, is another feasible alternative to treat the edentulous mandible. There are many merits for this procedure as it permits for longer implants placement. It also increases the contact area between bone and implant as well as the implant stability; it increases the distance between posterior and anterior implants which leads to enhancement the distribution of loads and it greatly reduces or eliminates the distal cantilever size. As a result, these benefits simplify the surgical procedure and reduce morbidity and time [7]. 

There are different options for correcting implant angulations such as angled abutments, angulated screw channels, or angulated prosthetic platforms built into the implant to allow for a screw retained crown. According to FEA, photo elastic stress evaluations, and strain gauge investigations, angled abutments have a negative impact on stresses transfer to prosthetic components and its surrounding bone [8, 9]. Angle-correcting implants fix the angle within the implant head rather than at the abutment level. The Co-Axis implant (Southern Implants) includes angulation correction options in the implant head of 12, 24, or 36 degrees, allowing implant insertion in areas with angled alveolar bone while the prosthetic platform is aligned to allow screw-retained restoration [10]. 

The continuous development in implants designs provided different solutions for the intended tilting of distal implants during following All-On-Four treatment protocol; either selecting implant system with angle correction design, at the fixture level like Co-Axis implants or angle correction design at the abutment level like using angled multiunit abutments. Which design is good according to stress distribution on the superstructure parts and the implant surrounding tissues was our investigation question.

Because finite-element analysis (FEA) simulation technique in the 3D space is a practical and dependable method, it can be used to assess stress and strain distributions in correlating prosthesis and implant outcomes [11–13]. Therefore, the goal of this study was to investigate, through FEA, the biomechanical behavior of the built-in angle corrected dental implants versus the angled multiunit abutments used in All-On-Four treatment protocol for mandibular fixed denture reinforced with a Ti mesh, regarding stress distributions around dental implant, implant abutments, denture framework and surrounding bone.

The null hypothesis states that there is no variation in stresses transferred to implant connections or to the surrounding alveolar bone when using the Angle -correcting implants or using multiunit angled abutments in managing intended implants angulation in All-On-Four screw retained full arch prosthesis.

## Materials and methods

### Model design

The existing FEA was intended to mimic the current clinical situation and to show the actual stresses and deformations that occurred on the prosthetic and proposed implants in the study. Based on the true dimensions, a fine 3D CAD model design was created, and the recommended material properties were applied to each part to begin the finite element analysis procedure.

**Fig. 1:**
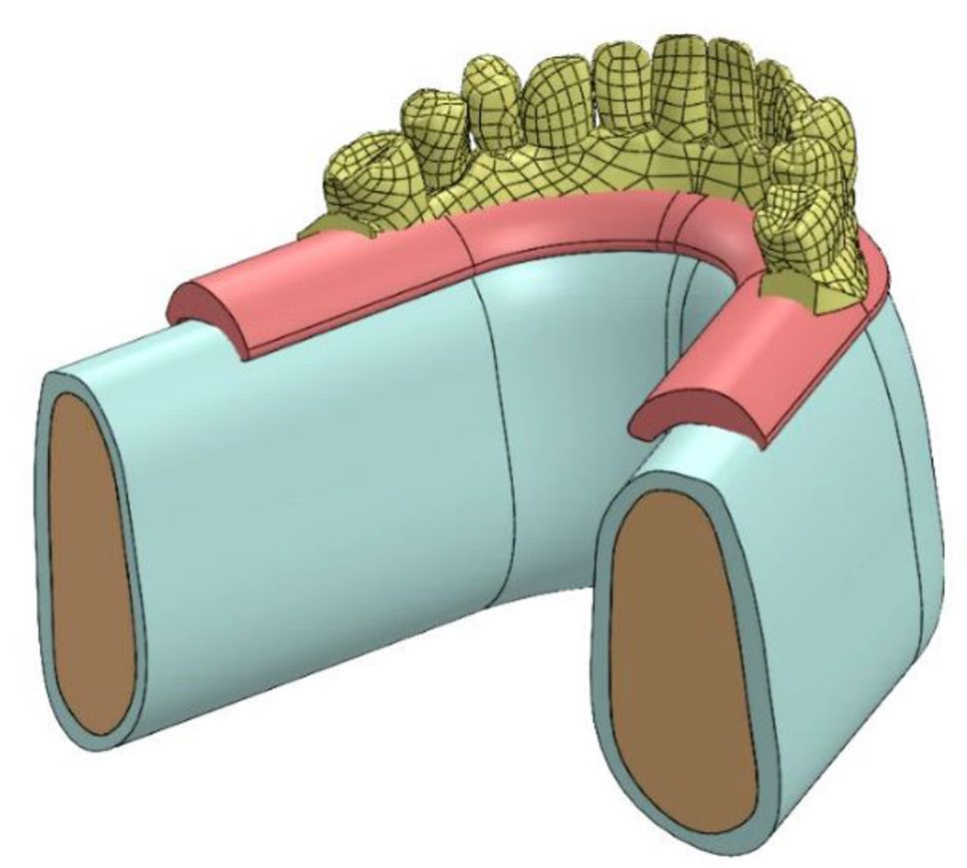
3D CAD Modeling of the proposed geometry

A 2-mm continuous cortical bone layer surrounded a cancellous bone core (19.2 mm) covered by a 2-mm-thick mucosa was in the suggested 3D virtual CAD model of the edentulous mandible [13, 14]. . For the modeling of a screw-retained fixed prosthesis, a Ti mesh in accordance with the implant and a superstructure with a wraparound acrylic denture base and 12 acrylic resin denture teeth were designed. The implants were modeled based on the **Co-axis southern implants** (4 mm diameter /15 mm length) and **Bredent Ski implants** (4 mm diameter / 14 mm length). Solidworks© release 2017 CAD software from Dassault Systems Company was used to model the desired geometry as shown in Fig. 1.

**Fig. 2:**
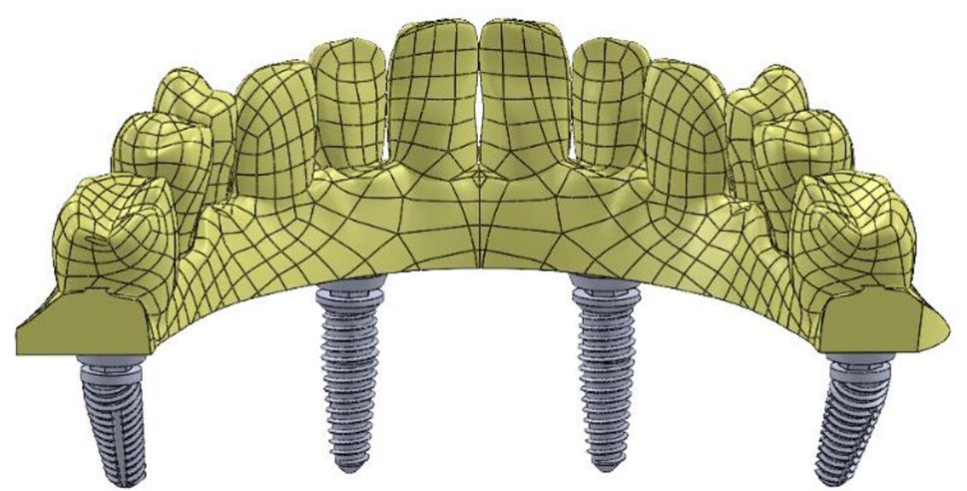
3D CAD Modeling of the Ti-mesh teeth frame with four proposed implants

**Fig. 3:**
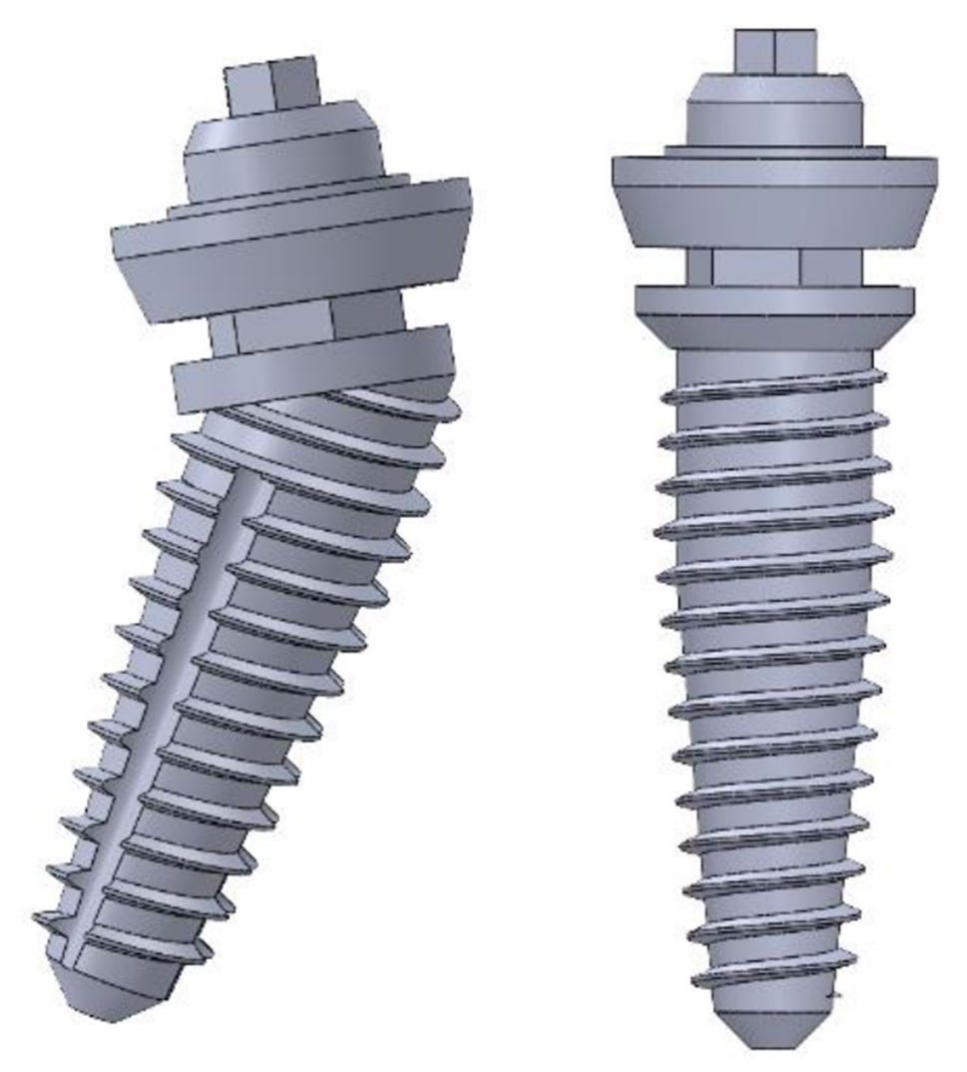
3D CAD model of Implants of Model I

**Fig. 4:**
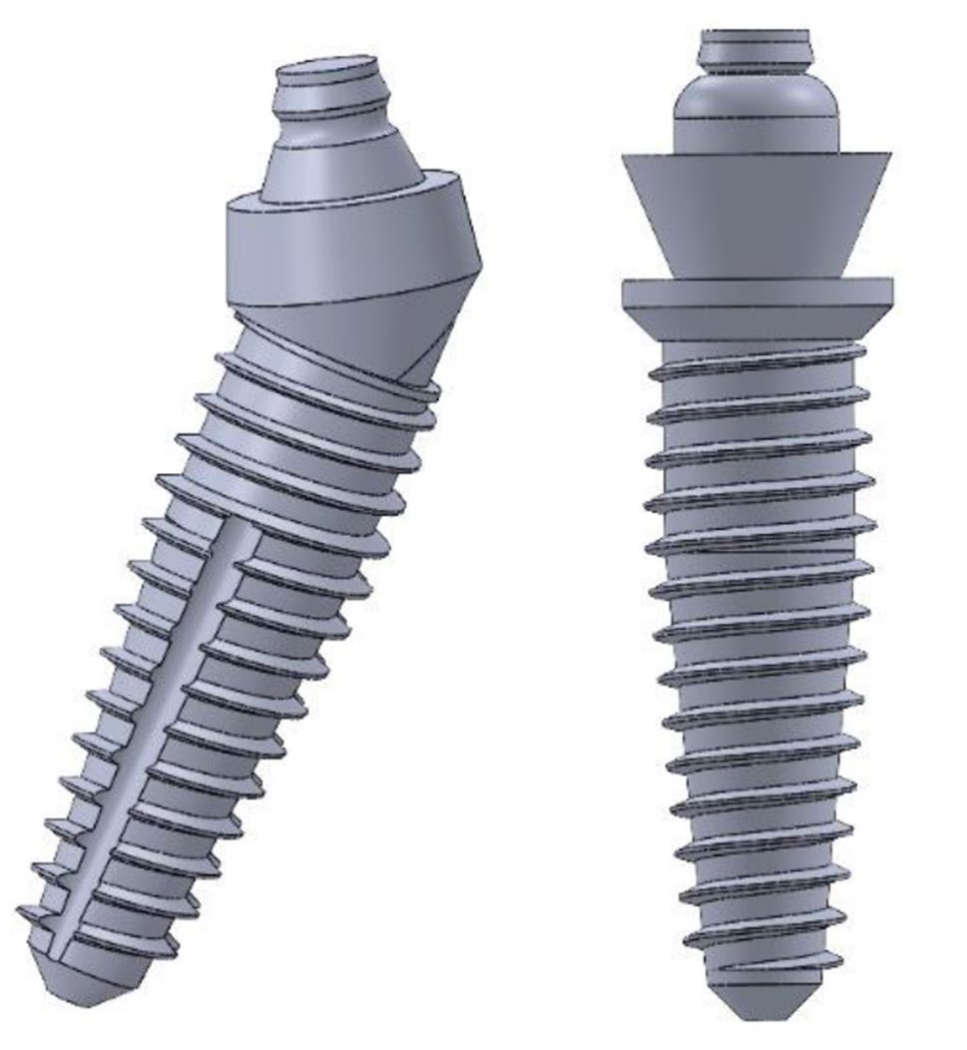
3D CAD model of Implants of Model II

Two CAD models with FEA preparations as simplified edentulous mandibles were constructed with two different posterior implant designs based on the All-On-Four protocol. Each model has four implants, two of them were anterior and placed vertically positioned at the lateral incisor/canine sites as shown in Fig. 2. Depending on the implant fixture design in posterior area, there are two models created; in Model I, the mandible was rehabilitated with four co-axis (4 mm in diameter × 15 mm in length) implants with distally built-in angle corrected implants (24-degree angle correction) and with conical abutments to support a screw retained mandibular fixed prosthesis with Ti mesh as shown in Fig. 3. While Model II, the mandible was rehabilitated with four conventional (4 mm in diameter × 14 mm in length) implants with a distally inclined posterior implants (25-degree) and angled multiunit abutments as shown in Fig. 4.The dimensions of the proposed CAD model of the edentulous jaw are 2.2 cm height, 1.8 cm width and 13.5 cm length and the model were built using Solidworks©. All implants and abutments were modeled based on the actual dimensions from the commercial data sheets of each manufacturer.

### Finite element analysis

**Fig. 5:**
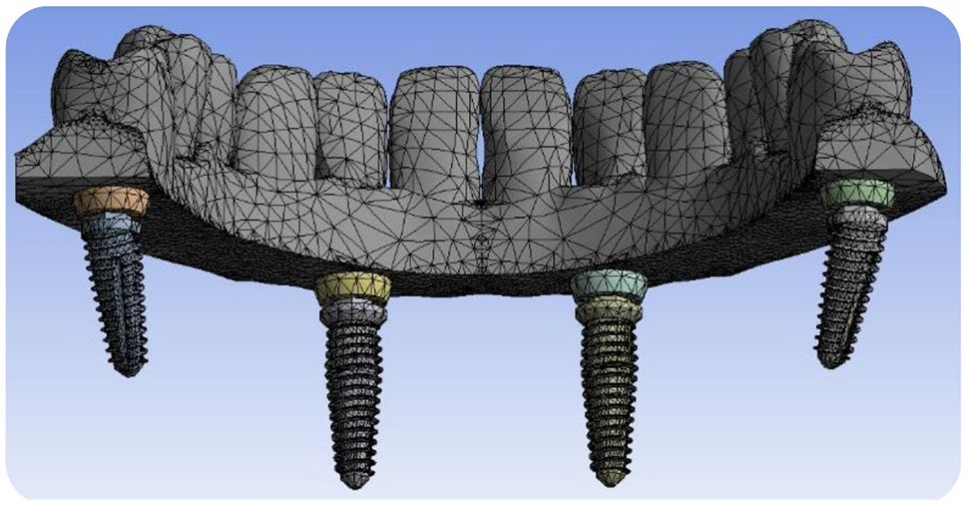
Meshing process of the proposed Model

After that, the FEA step started by importing the models with all parts to an efficient 3D analysis software Ansys© 2019 from Ansys Company. Then, the materials used for each part were selected, taking into account the specifications and determination of their properties with great precision, especially for those materials that are not included in the program, such as cortical, cancellous bones, and other composite materials as reported in Table 1. The meshing process is the next step to apply meshing characteristics to all geometry regions of the proposed model as shown in Fig. 5. The necessary load conditions were applied to the model parts at the selected positions according to the study requirements.

**Table 1:** Properties of materials used for finite element analysis models

Material	Density (Kg/m^3^)	Young’s Modulus (MPa)	Poisson’s Ratio	Tensile Yield Strength (MPa)	References
Titanium	4400	110,000	0.35	834	[22, 23]
Mucosa	1400	0.34	0.45	4	[20, 23]
Acrylic resin denture base	1190	2700	0.3	61	[20, 23]
Acrylic resin teeth	1190	2940	0.3	61	[20, 23]
Cortical bone	1990	13,700	0.3	114	[20, 23]
Cancellous bone	1847	1370	0.3	52	[23, 24]

Model I had 211,131 elements and 356,544 nodes; Model II had 157,390 elements and 277,136 nodes with mesh convergence check for the proposed model and applying automatic adaptive meshing technique in ANSYS. The meshing element type is tetrahedron ten nodal element. Table 1 shows all materials used in the proposed study with all its mechanical characteristics needed in our study. All these properties were included in Ansys software library before starting the simulation analysis. The bone tissues were thought to be isotropic, linear, homogeneous, and completely osseointegrated with the implants. To simulate virtually with conditions similar to real clinical conditions, all the components were assumed to exhibit bonded contact except for the interface between the bone and the implants. Slip (no-penetration) contact interface between the bone and the implants was defined with a friction coefficient of 0.3 to simulate immediately loaded implants [15–17]. 

**Fig. 6:**
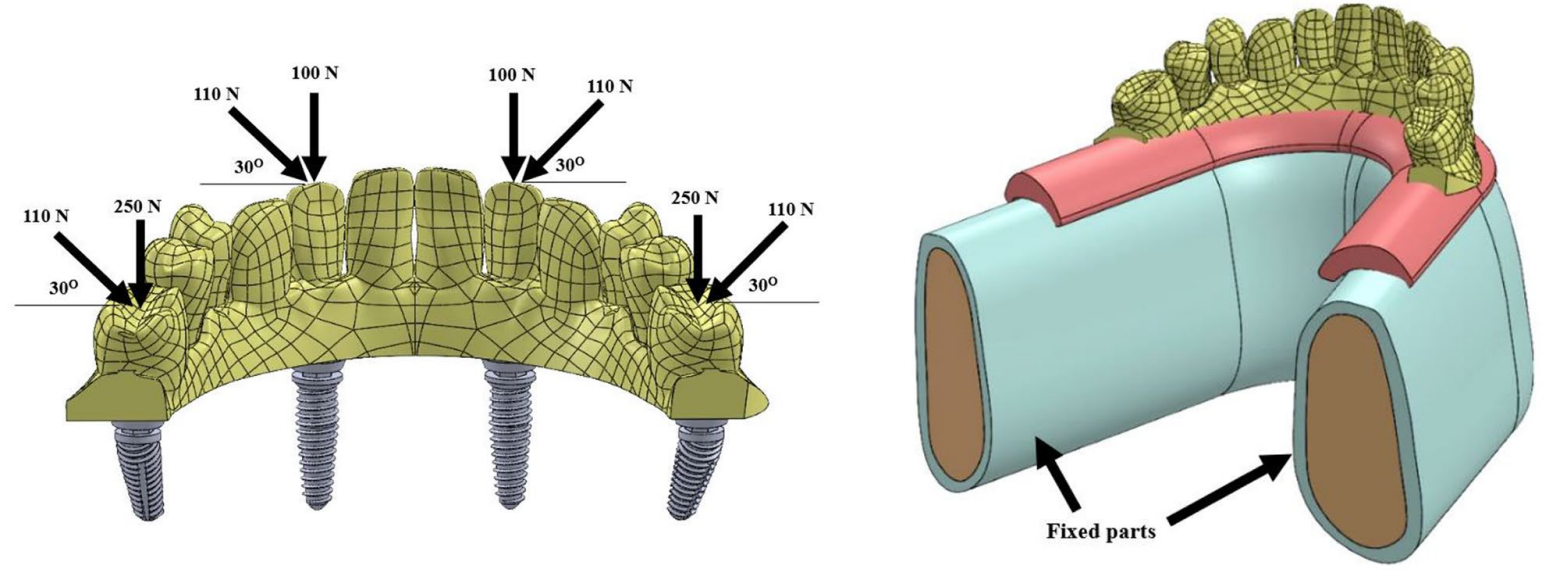
3D CAD model showing loading conditions and fixed parts

A **100-N axial** load was applied on anterior implants with **250-N axial** load applied bilaterally and simultaneously on the distobuccal and mesiobuccal ends of the **first molar** in the posterior region [18]. To replicate the mean value of the occlusal load, a load of **110-N** is applied to the **first molar** (30 degrees of buccolingual direction, inclination about the long axis of the implant). Besides, to mimic lateral mandibular movement, oblique load of **110-N** is applied on canine area (angled 30 degrees) [19] as shown in Fig. 6. The prosthesis and implant configurations for both models were analyzed, and the stress distribution and deformation analysis were obtained for each part (implants and prosthetic components). By using the stress distribution map, we can identify regions with maximum stresses and deformations.

Also, we can predict the performance of the implant and the loads applied to it according to the maximum values of displacement resulted. Also, we can simulate the maximum principal stresses and stress intensity to know about the strength of each part of the proposed model and to be able to predict failure in the future in any part in the proposed assembly.

## Results

The region of interest in our study is the effect of diverse implant designs on stress distribution in a screw retained full arch mandibular prosthesis and surrounding supporting bone according to All-On-Four placement protocol. Also, the results will help us to select the best dental implants’ design that can sustain the applied loads with reasonable life-time and reasonable strength. A detailed discussion will be presented after showing all the results. The results of the finite element analysis of the proposed model will include calculating and simulating **total deformation**, **equivalent Von-Misses stresses**, and **maximum principal stresses** as follows:

### Deformation analysis

**Fig. 7:**
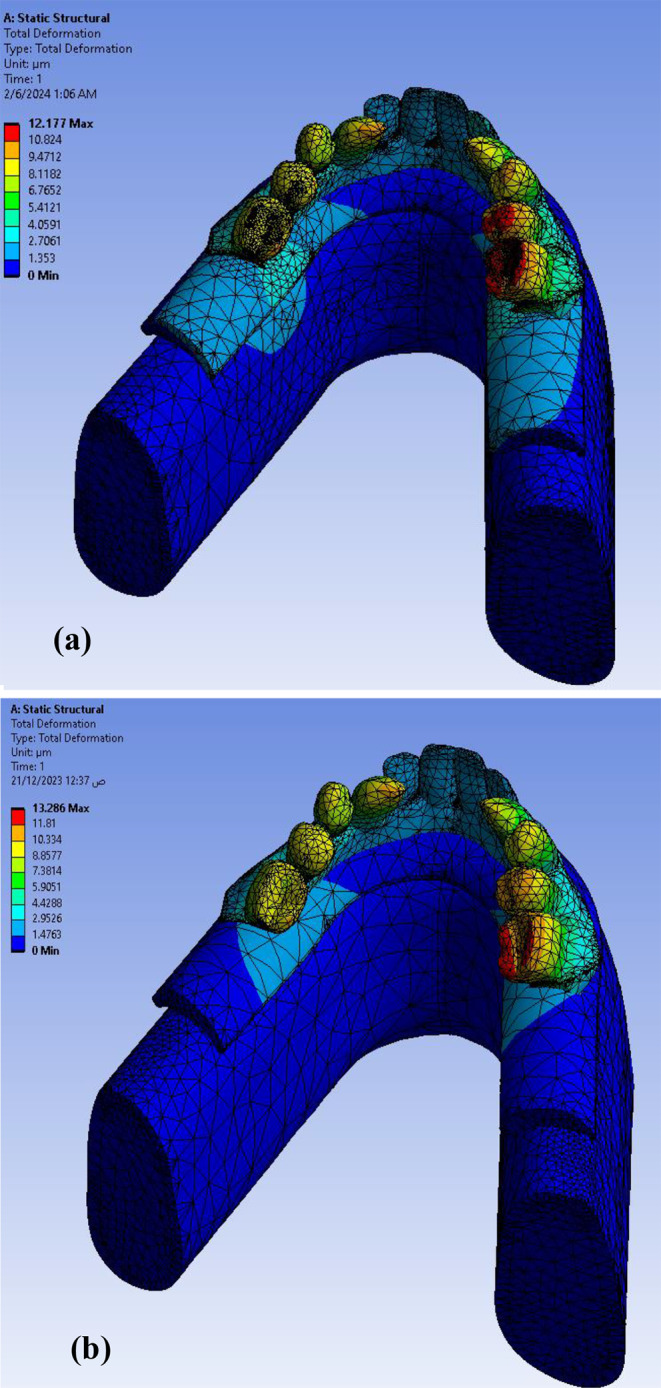
Total deformation in full assembly under loading conditions in both models (**a**) Model I (**b**) Model II

Calculating the deformation (displacement) that occurs in the proposed model is considered one of the most important steps to determine the extent of the body’s ability to resist the change that occurs to it due to the influence of external forces. After applying the meshing characteristics and loading conditions (axial and non-axial) the maximum total deformation of the proposed model (full assembly with all parts) was found to be as **12.177** μm for **Model I** and **13.286** μm for **Model II** as shown in Fig. 7. So, **Model II** was more deformed than **Model I** but not statistically significant as verified by Mann Whitney test (P value (0.317).

**Fig. 8:**
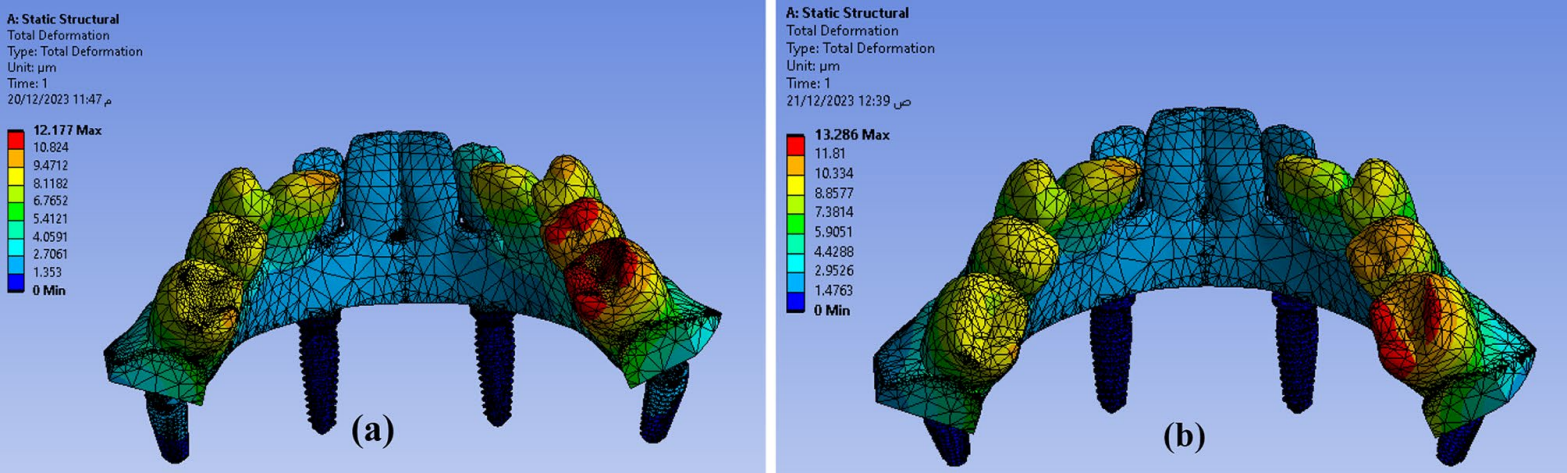
Total deformation in Ti mesh in both models: (**a**) Model I (**b**) Model II

**Fig. 9:**
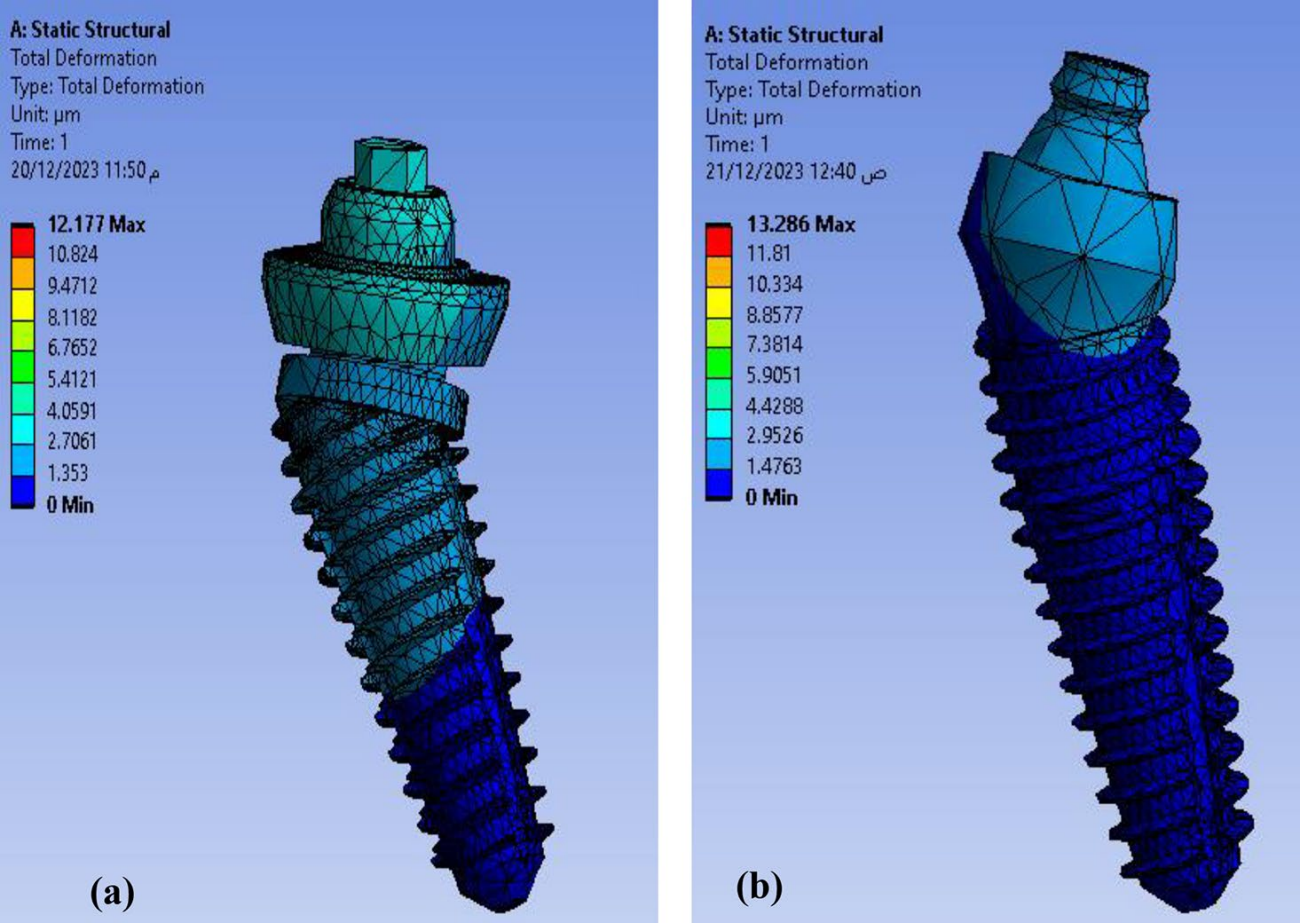
Total deformation on posterior implants in both models (**a**) angle corrected posterior implant in Model I (**b**) Inclined posterior implants with angled multiunit abutment in Model II

**Fig. 10:**
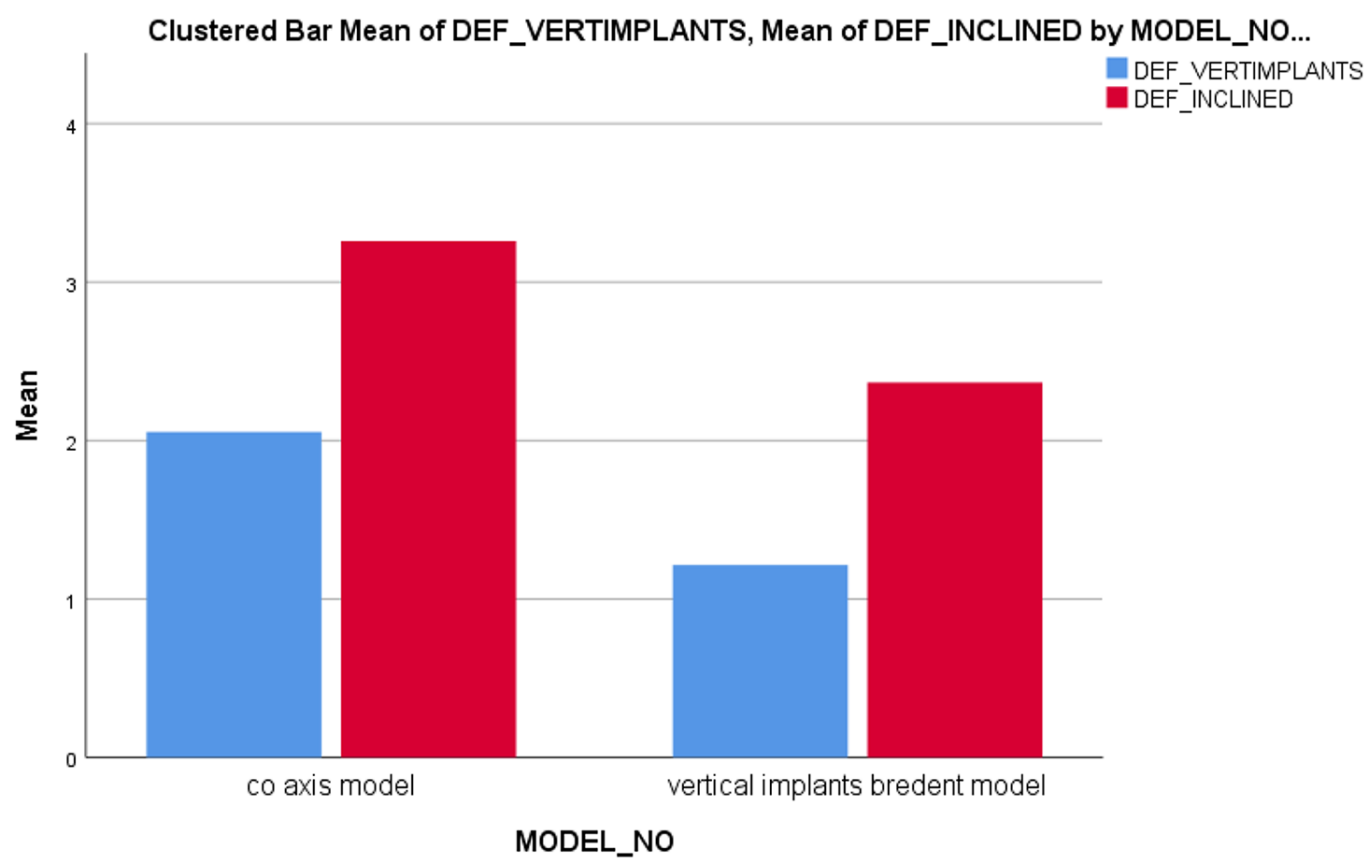
Deformation applied on vertical and inclined implants (All-On-Four concept) in both models Co-axis model as **Model I** and Bredent model as **Model II**

The Ti mesh was highly deformed with a value of about **13.286** μm in **Model II** than the **Model I 12.177** μm as shown in Fig. 8. The maximum deformation on posterior implants in both models were calculated precisely as angle corrected posterior implant in **Model I** was more deformed with a value of about **3.2589** μm in implant abutment than inclined posterior implants with angled multiunit abutment in **Model II** as **2.366** μm but not statistically significant as verified by Mann-Whitney test (P value 1.00) as shown in Fig. 9. In both models, the inclined implants are more deformed than vertical anterior implants. The mean displacement in µm between anterior and posterior implants in each model is shown in Fig. 10, it showed that deformation on posterior inclined implants **3.2589 Model I** µm and **2.366** μm **Model II** respectively was more than deformation on anterior vertical implants **2.0536** μm **Model I** and **1.2136** μm in **Model II** respectively. However, there was no statistically significant difference in the deformation between **Model I** and **Model II** when axial and non-axial load applied.

**Fig. 11:**
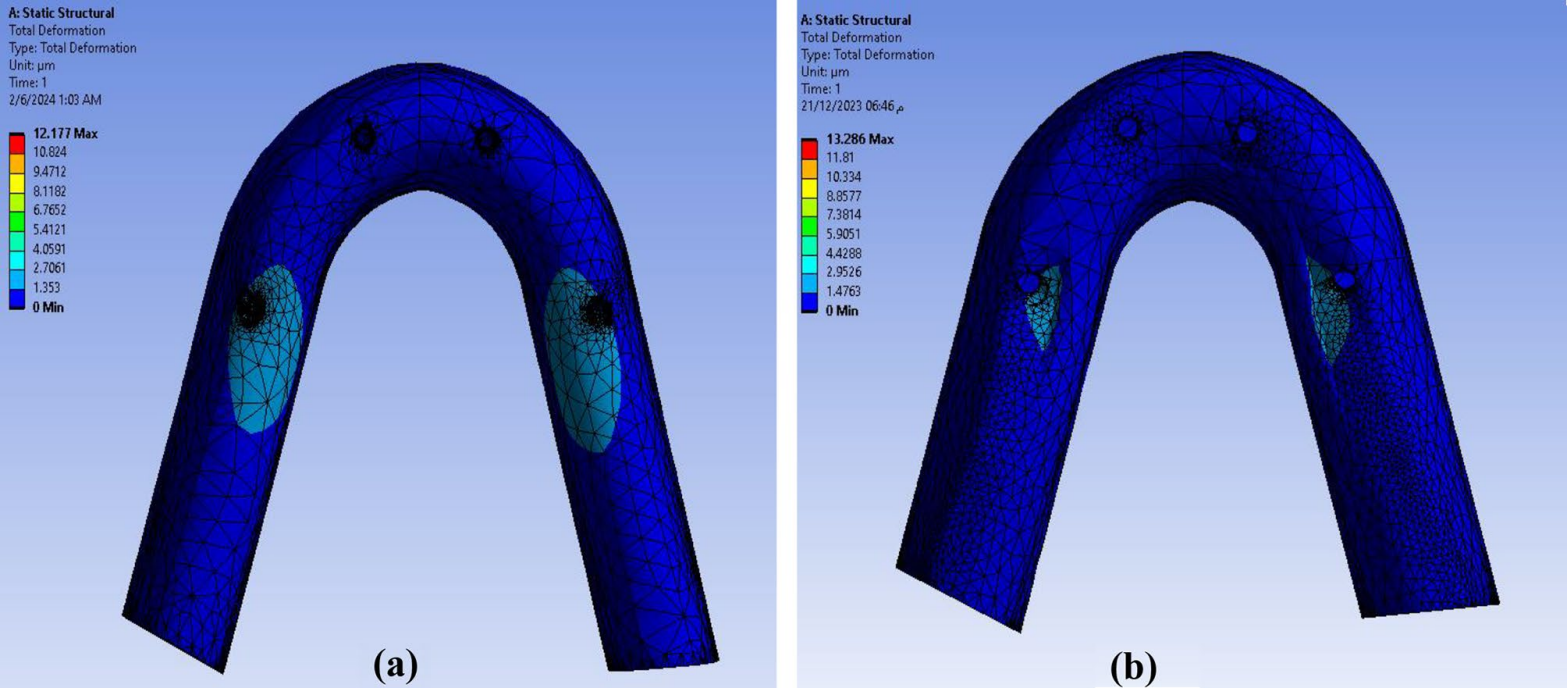
Total deformation on the peri-implant bone in both models (**a**) Model I (**b**) Model II

The maximum deformation at peri-implant bone was higher at **Model I** with a value of **2.8469** μm than **Model II** as **2.0723** μm but the difference was not statistically significant verified by Mann Whitney test P value (0.317) as shown in Fig. 11.

### Von-misses stresses (VMS)

Von-Misses stress criterion is used widely to figure out the yielding condition for ductile materials. This theory is giving us a great impression about the sustainability of material under loading conditions.

**Fig. 12:**
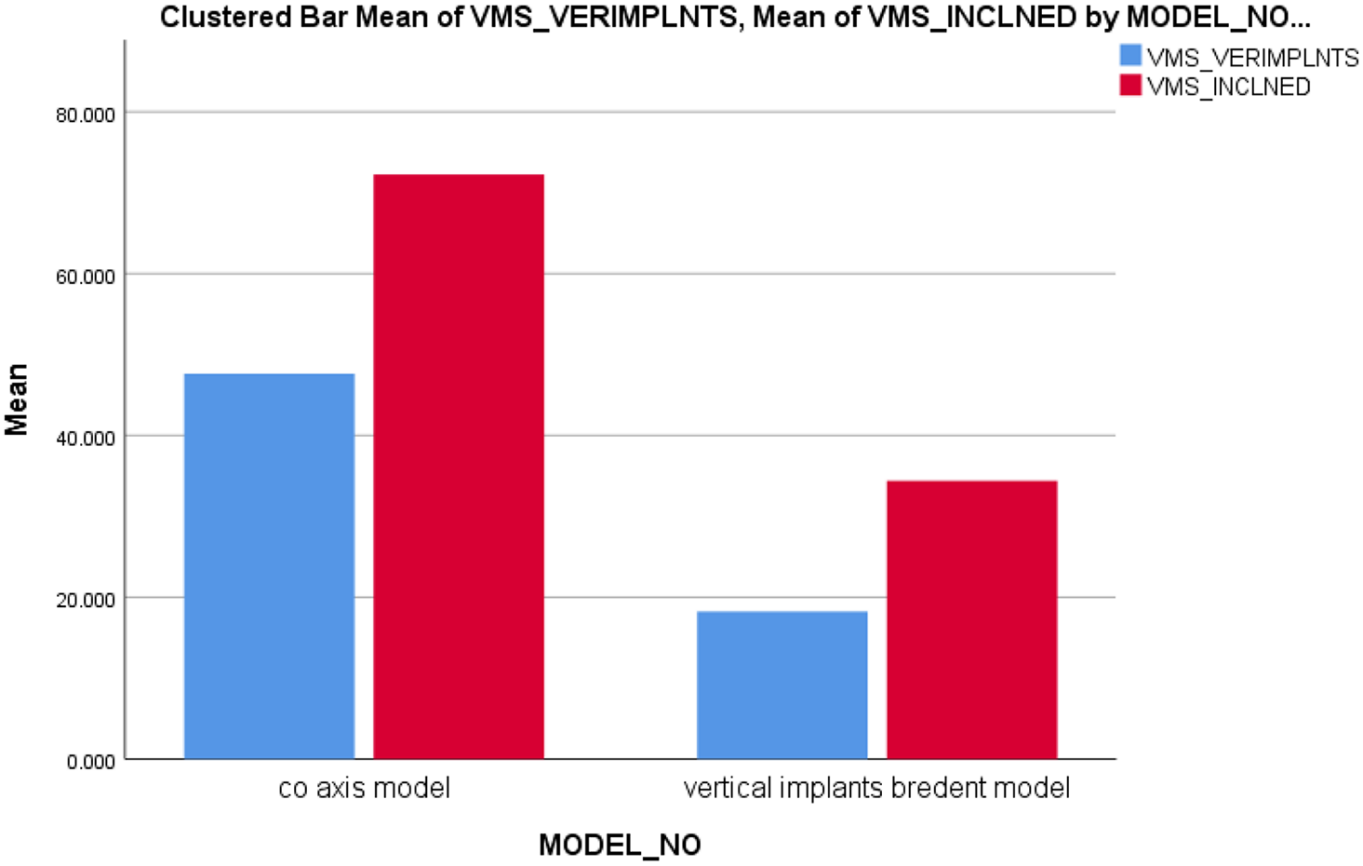
Von-Misses stresses applied on vertical and inclined implants (All-On-Four concept) in both models Co-axis model as **Model I** and Bredent model as **Model II**

The equivalent Von-Misses stress values were calculated and simulated precisely using ANSYS software as follows:

As shown in Fig. 12, the maximum VMS in Mega Pascal (MPa) on implants in both models were presented. For posterior inclined implants, stresses were slightly higher in **Model I** with a value equal to **72.247** MPa than **Model II 34.386** MPa but not statistically significant. For anterior vertical implants, VMS were higher in **Model I** as **47.631** MPa than **Model II** with a value equal to **18.235** MPa but the results also were not significant as verified by Mann Whitney test.

**Fig. 13:**
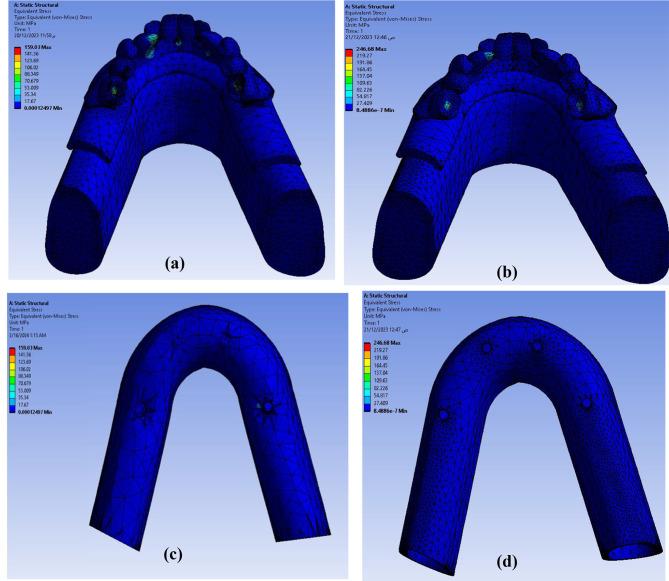
Equivalent Von Misses stresses distribution (**a**) full assembly of Model I, (**b**) full assembly of Model II, (**c**) peri-implant bone of Model I, (**d**) peri-implant bone of Model II

**Fig. 14:**
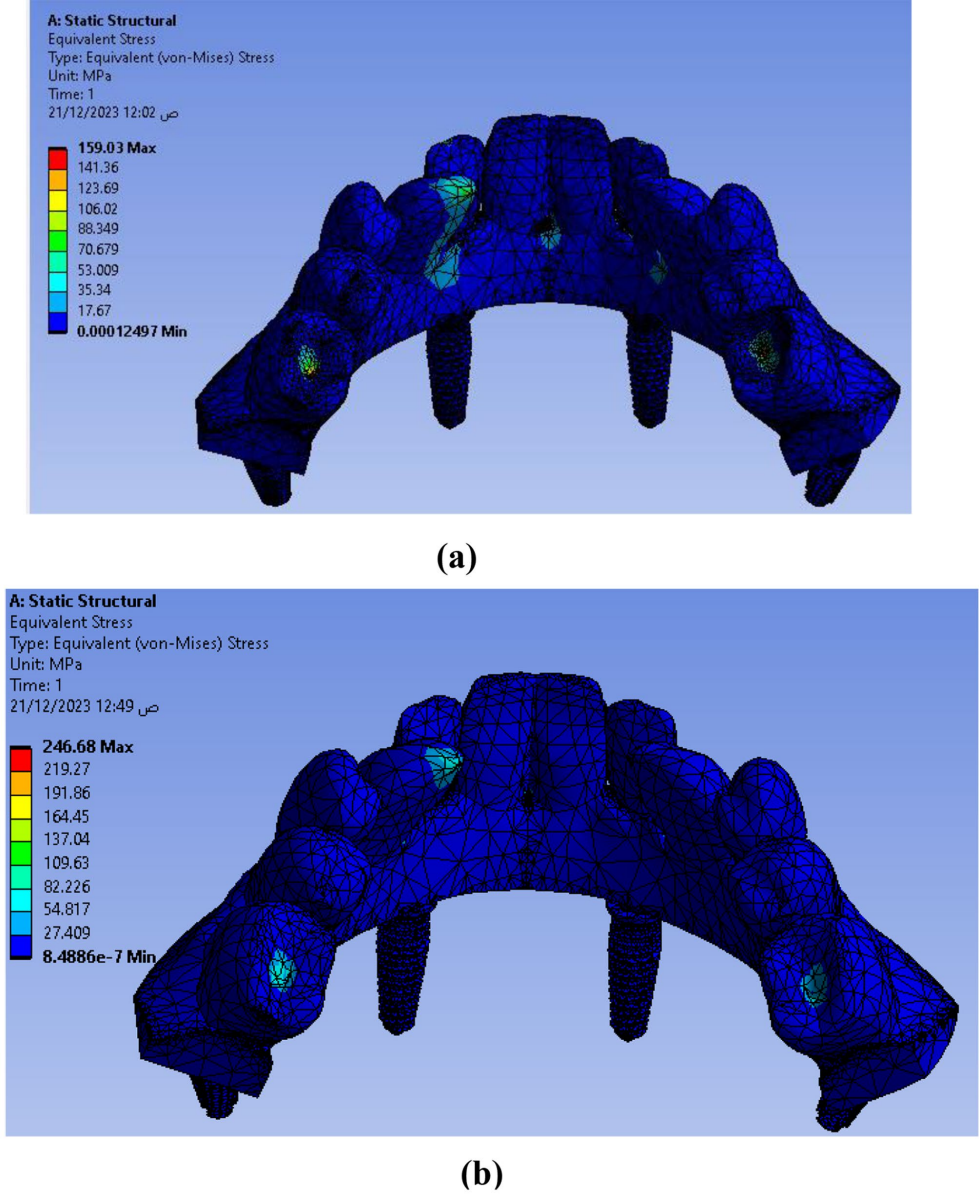
Equivalent Von Misses stresses distribution on Ti mesh with implants (**a**) Model I, (**b**) Model II

Figure 13 shows the equivalent VMS distribution for both models as follows: **Model II** discloses an abundant stress concentration (**246.68** MPa) in the lingual side of anterior teeth specially the canine tooth and in the center of last molar tooth on both sides than **Model I** (**159.03** MPa). For peri-implant bone, the maximum VMS was lower in **Model II** (**3.542** MPa) than of **Model I** (**4.631** MPa). Figure 14 shows the maximum (VMS) on TI mesh in (MPa) that was higher in **Model II** than **Model I**. However, the difference was not statistically significant.

### Maximum principal stress

**Fig. 15:**
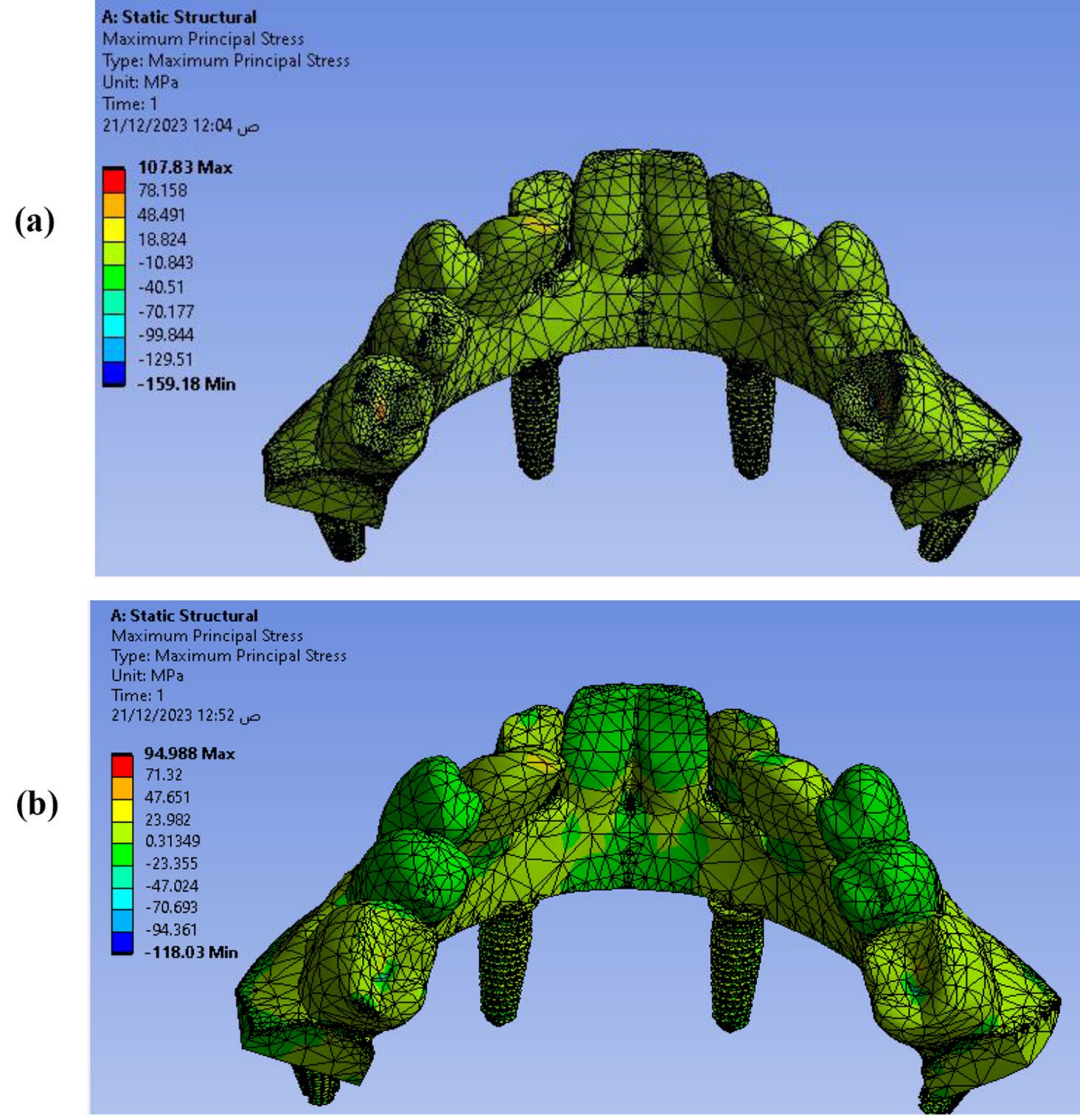
Maximum principal stresses distribution on Ti mesh with implants (**a**) Model I, (**b**) Model II

**Fig. 16:**
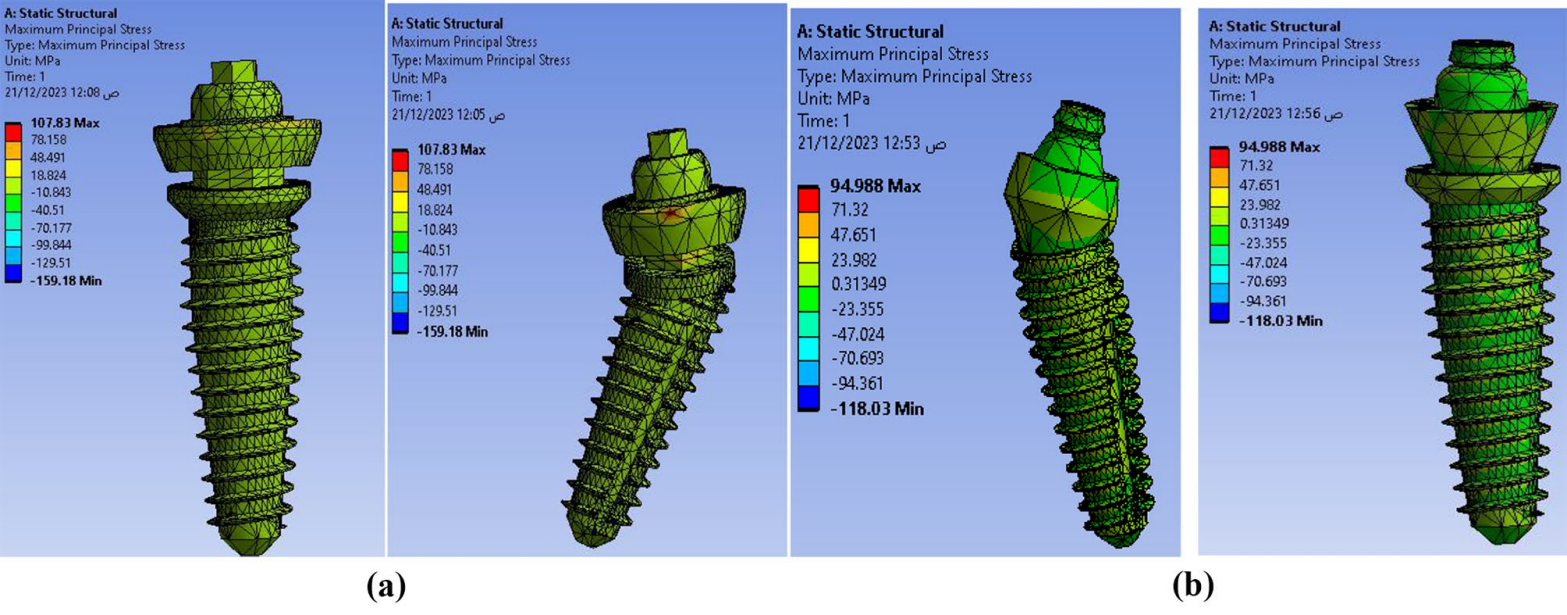
Maximum principal stresses distribution on implants (**a**) Model I, (**b**) Model II

The maximum principal stress is used to monitor the effect of external payloads on material that has to some extent brittle characteristics, and it can be used to show the rigidity of the model parts. The maximum principal stresses were calculated in MPa for the proposed models as **107.83** MPa for **Model I** and **94.988** MPa for **Model II** for Ti mesh with all four implants as shown in Fig. 15. The maximum principal stress value for anterior vertical implants is 33.037 MPa for **Model I** and 12.707 MPa for **Model II**. The values for posterior implants are 106.66 MPa for Model I which is higher than the **Model II** with a value equal to 12.319 MPa as shown in Fig. 16. So, Model I is showing higher values compared with Model II in maximum principal stresses which affect lifetime of the proposed model strength and rigidity.

## Discussion

The manner in which stresses are passed to the surrounding bone is one of the most crucial deciding elements in the success or failure of a dental implant and restoration. Based on the previous results we can state that the difference in deformation and VMS between both models was not significantly different and the null hypothesis was accepted.

The Ti-mesh was highly deformed about 13.286 μm in (Model II) than the (Model I) 12.177 μm when applying axial and non-axial forces. This may be due to presence of more screw connections in case of using multiunit abutments in model (II) (screw between implant fixture and angled MUA, and another one between MUA and TI meshwork). This will increase the vertical lever arm than Model I accompanied with more deformation under axial and non-axial loading. This was in a line with Huang et al. 2019 who described that the process of tightened and elongation of the screws of a screw-retained restoration within the elastic range, creating a tensile force called the preload. The preload determines the clamping force exerted by the screw between the joined parts, holding them together [20]. 

So, under axial and non-axial load like applied in this study, external forces decrease the elastic deformation of the screw, and the clamping force is decreased with increase in the micromotion at the implant –abutment interface leading to increase in the displacement and deformation of the Ti meshwork due to screw loosening [20, 21]. 

In Both models, the inclined posterior implants were more deformed than vertical anterior implants. The posterior inclined implants were deformed 3.2589 (model I) µm and 2.366 μm (model II) respectively.This was in agreement with several clinical studies which reported that increase posterior implants angulation by 15° associated with increased stresses by 21 Mega Pascal (MPa) compared with anterior straight ones [22–26]. . Moreover, the increase of deformation in inclined posterior implants may be due to the location of inclined posterior implants which were closest to the loading area. Shear forces are increased with increasing the implant inclination as suggested by Sannino G [27].

The maximum deformation was at posterior peri-implant bone. It was higher at model (I) (2.8469 μm) than model (II) (2.0728 μm) but the difference was not statistically significant. This may be related to a smaller number of screw connections in model (I) design which results in more transfer of the stresses to the surrounding bone. On contrary, model (II) has two screw connections design; between the abutment and the (MUA) and between the (MUA) and the implant fixture, which may result in reduction of some of the stress before reaching to the surrounding alveolar bone.

This finding was consistent with several clinical studies by Huang el al [20]. , Asvanund el al [28]. , Hein et al. [29] and El-Sheikh et al. [30] Who reported that increasing abutment angulation in case of using MUA can increase the loss of preload under cyclic loading which contributes to dissipation of some of the stresses passed to the surrounding bone as in Model II. Also, the fact that model (II) has abutment connection at abutment level of the multiunit abutment can increase the vertical cantilever arm. This was in accordance with Asvanund P et al. 2011 who found that the connection proximity to the implant center decreased the vertical lever arm [28].

Von-Misses stress (VMS) is a combination of normal and shear stresses which predicts the yielding of materials under complex loading. In this study, it revealed that, model (II) showed non-significant higher stresses in the posterior inclined implants compared to the stresses in the posterior inclined implants of model (I). This was in agreement with several studies which reported an increase in VMS on inclined posterior implants in All-On-Four protocol than straight anterior implants as they explained this by the more inclination angle the more increase in the shear forces on posterior implants [22, 31]. 

Interestingly, model (I) anterior implants showed higher VMS stress by 31.309 MPa compared to 15.972 MPa in model (II) which may be due to the ability to share more load to the anterior implants in model (I) than model (II) due to the presence of external implant connection in model (I). This was consistent with photo-elastic stress study by Asvanund et al. 2011.who found that when the prosthesis was loaded anteriorly and unilaterally, the external-implant abutment connection generated more stresses at the implant-abutment connection level than the internal-implant abutment connection [28]. 

For peri-implant bone VMS, the maximum VMS was lower in model (I) (2.47 MPA) than of model (II) (10.3 MPA). This may be due to the rigidity of the design in abutment framework connection. This finding was found to be in agreement with the conclusion that was reported by kelkar et al. 2021 [32] as the more rigid framework material showed minimal stress distribution in all parameters at implant–bone interface compared with the less rigid PEEK framework materials. As the rigid framework materials may themselves absorb higher stresses than flexible ones as reported with Tribst et al. leading to less stresses would dissipate to the implant bone interface [33]. The use of rigid framework material clinically would thus prevent failure of the implant support system.

Model I is showing higher values compared with Model II in maximum principal stresses specially at posterior implants in both models. This was in agreement with a study by Boukhlit et al. 2020 [34], the authors reported a decrease in maximum principle stresses from 90.04 MPa to 46.36 MPa by increasing the implant fixture inclination degree Thus, a rise in inclination has suitable effects on stress distribution pattern and can be optimized for better results .

Another finite element study by Mohamed et al. 2021 [35] found that an abutment angulation 24 degrees angle will make the fixture situation more critical than before. Besides, the 21 degrees abutment puts the fixture in a more critical condition, making it more likely to become plastic early. For abutment screws, it showed different conditions than fixtures. Because with the increase of the abutment angle, the stress distribution in the screws has decreased more and more each time which explained why Model II is lower than Model I.

Lastly, all models used in this study were considered to be homogeneous, isotropic, and linear elastic. Actually, there is no 100% homogeneous and isotropic material in nature. In this case, assuming that the material is homogeneous and isotropic, the use of mean values does not preclude the in vitro test results to be close to the original [25]. 

Moreover, it was also assumed that the connection between implants and bone is 100%. It is a known fact that there is never 100% connection between bone and implant [22]. These factors are limitations of our study. For this reason, it is necessary to take into account the limitations of the finite element stress analysis method when evaluating the results of the study [31]. 

## Conclusion

Within the limitation of the FEA study, although angle correcting implant design is showing higher values in maximum principle stresses compared with angled multiunit abutments, model deformation and resultant VMS increased with angled multiunit abutments. The angle correcting designs at implant level have more promising results in terms of deformation and VMS distribution than angle correction at abutment level.

*In clinical practice, the use of angle correcting designs at implant level has promising results in terms of Framework deformation and VMS than correcting the angulation at abutment level that need to be confirmed by more clinical studies.*

### Electronic supplementary material

Below is the link to the electronic supplementary material.

Supplementary material 1: Deformation for model I

Supplementary material 2: Deformation for model II

## Data Availability

The datasets used during the current study are available from the corresponding author on reasonable request.
